# ALCAM/CD166 Is Involved in the Binding and Uptake of Cancer-Derived Extracellular Vesicles

**DOI:** 10.3390/ijms23105753

**Published:** 2022-05-20

**Authors:** Beatriz Cardeñes, Irene Clares, Tamara Bezos, Víctor Toribio, Soraya López-Martín, Almudena Rocha, Héctor Peinado, María Yáñez-Mó, Carlos Cabañas

**Affiliations:** 1 Centre for Molecular Biology “Severo Ochoa” (CSIC-UAM), Cell-Cell Communication & Inflammation Unit, 28049 Madrid, Spain; bea_car@hotmail.com (B.C.); ireneclares@gmail.com (I.C.); tamarabezoscarmona@hotmail.com (T.B.); victor.toribio@estudiante.uam.es (V.T.); slopez@cbm.csic.es (S.L.-M.); almrocha@ucm.es (A.R.); maria.yannez@uam.es (M.Y.-M.); 2 Department of Molecular Biology, Faculty of Sciences, Instituto Universitario de Biología Molecular, Universidad Autónoma de Madrid, 28049 Madrid, Spain; 3 Microenvironment and Metastasis Laboratory, Molecular Oncology Programme, Spanish National Cancer Research Center (CNIO), 28029 Madrid, Spain; hpeinado@cnio.es; 4 Instituto de Investigación Sanitaria Hospital La Princesa, 28006 Madrid, Spain; 5 Department of Immunology, Ophthalmology and Otorhinolaryngology, School of Medicine, Universidad Complutense de Madrid, 28040 Madrid, Spain; 6 Instituto de Investigación Sanitaria Hospital 12 de Octubre (i+12), 28041 Madrid, Spain

**Keywords:** extracellular vesicles, intercellular communication, uptake, docking, colorectal cancer, ovarian cancer, ALCAM, CD166

## Abstract

Colorectal cancer (CRC) and ovarian cancer (OvC) patients frequently develop peritoneal metastasis, a condition associated with a very poor prognosis. In these cancers, tumor-derived extracellular vesicles (EVs) cause immunosuppression, facilitate the direct attachment and invasion of cancer cells through the mesothelium, induce the conversion of peritoneal mesothelial cells (PMCs) into cancer-associated fibroblasts (CAFs) and transfer a more aggressive phenotype amongst cancer cells. Although the promoting role of EVs in CRC and OvC peritoneal metastasis is well established, the specific molecules that mediate the interactions between tumor-derived EVs and immune and non-immune target cells remain elusive. Here, we employed the SKOV-3 (ovarian adenocarcinoma) and Colo-320 (colorectal adenocarcinoma) human cell lines as model systems to study the interactions and uptake of EVs produced by ovarian carcinoma and colorectal carcinoma cells, respectively. We established that the adhesion molecule ALCAM/CD166 is involved in the interaction of cancer-derived EVs with recipient cancer cells (a process termed “EV binding” or “EV docking”) and in their subsequent uptake by these cells. The identification of ALCAM/CD166 as a molecule mediating the docking and uptake of CRC and OvC-derived EVs may be potentially exploited to block the peritoneal metastasis cascade promoted by EVs in CRC and OvC patients.

## 1. Introduction

Different types of EVs, including exosomes, are released by virtually all cell types and may work as vehicles of intercellular communication through the transfer of their biomolecular cargo of proteins, nucleic acids (mRNAs, miRNAs, DNAs) and lipids between donor and recipient target cells [1,2,3].

EVs released by malignant cells play key roles in tumor development, progression and metastasis in many types of cancer, including colorectal (CRC) and ovarian (OvC) carcinomas [4,5,6]. CRC and OvC frequently spread (in the case of OvC, almost exclusively) from the primary tumor through local invasion of neighboring abdominal structures and subsequent peritoneal dissemination [7,8,9]. Patients of CRC and OvC often develop malignant ascites, an increased amount of peritoneal fluid that is induced by the tumor through leakiness of the microvasculature and the obstruction of lymphatic vessels [7,10,11]. Malignant ascites represents an advanced stage of disease and is associated with the worst prognosis. Tumor-derived EVs are very abundant in the malignant ascites of CRC and OvC patients and have been shown to promote peritoneal metastasis through their specific interaction with—and uptake by—different immune and non-immune target cells in the peritoneal environment, in which they induce crucial phenotypic and functional changes involved in tumor progression and metastasis. In this regard, tumor-derived EVs cause immunosuppression and facilitate the direct attachment of cancer cells to peritoneal mesothelial cells (PMCs) that line all peritoneal structures, thus promoting the invasion of cancer cells through the mesothelium and the conversion of PMCs into cancer-associated fibroblasts (CAFs), which in turn, favor cancer-cell survival, proliferation, and invasion (reviewed in [12,13,14,15]). Importantly, tumor-derived EVs also mediate communication amongst cancer cells themselves. In this regard, it has been shown that EVs produced by cancer cells can transfer a more aggressive and invasive phenotype to other cancer cells, as well as conferring resistance to chemotherapy treatments [16,17,18,19]. Although all these promoting roles of EVs in CRC and OvC peritoneal metastasis have been relatively well established, knowledge of the specific molecules that are involved in the plethora of interactions between tumor-derived EVs and their immune and non-immune target/receiver cells is at present rather scarce.

Activated Leukocyte Cell Adhesion Molecule (ALCAM/CD166) is a 100–105 kDa type-I transmembrane glycoprotein that belongs to the immunoglobulin superfamily (Ig-SF) of adhesion receptors. ALCAM contains five extracellular Ig-like domains (V-V-C2-C2-C2, in the Ig-SF nomenclature) in its extracellular portion, one transmembrane region and a short cytoplasmic tail, and mediates cell–cell adhesion phenomena through either homophilic (ALCAM-ALCAM) or heterophilic (ALCAM-CD6) interactions with its ligand CD6. Functional studies have implicated ALCAM-mediated adhesion in a variety of crucial physiological and pathological processes. Since the ligand CD6 is an “accessory molecule” and signal-transducing receptor that is almost exclusively expressed by lymphocytes, heterophilic interactions between ALCAM on antigen-presenting cells (APCs) and CD6 on T lymphocytes have been shown to be essential for immunological synapse stabilization and optimal T cell activation and proliferation [20,21,22]. However, in contrast to CD6, ALCAM is widely distributed in many tissues and its expression seems to be restricted to specific subsets of cells involved in dynamic growth and migration processes [23]. Accordingly, ALCAM mediates leukocyte transendothelial migration and trafficking into the central nervous system [24,25,26] and the growth, progression and metastasis of many different tumors, including colorectal and ovarian carcinomas (reviewed in [26,27,28,29,30]).

The ectodomain of transmembrane cellular ALCAM can be cleaved by the metalloproteinase ADAM17/TACE (A Disintegrin and Metalloproteinase-17/Tumor Necrosis Factor α Converting Enzyme), resulting in the release of a soluble form of ALCAM (sALCAM), a process termed “ectodomain shedding” [31,32,33]. In colorectal cancer, high ALCAM shedding is associated with cancer progression and correlates with poor patient outcomes, being significantly increased in stage II, III, and IV patients [34]. Likewise, ALCAM shedding by ADAM17/TACE is involved in the motility of OvC cells and sALCAM can be detected in the serum and malignant ascites of OvC patients [35]. Furthermore, sALCAM has been proposed as a potential biomarker of epithelial OvC and elevated sALCAM seems to be an early feature of aggressive epithelial OvC [35]. On the other hand, full-length transmembrane ALCAM was found on tumor-derived peritoneal EVs in OvC [35], although the involvement of this exosomal ALCAM in the pathology of OvC has not been established.

Previously, our group employed the SKOV-3 (ovarian adenocarcinoma) and Colo-320 (colorectal adenocarcinoma) human cell lines as useful models to study cancer-related processes (including tumorigenesis, metastasis, and tumor cell adhesion), as well as the regulatory functions and interplay between tetraspanins and cell adhesion molecules [36,37,38]. In the current research, we used these cells lines as a model system to study the interactions of EVs produced by OvC (SKOV-3) or CRC (Colo-320) cells with recipient cancer cells and with peritoneal mesothelial cells. Our results show that the adhesion molecule ALCAM/CD166 participates in the “binding/docking” of cancer-derived EVs and in their subsequent uptake by recipient cancer and peritoneal mesothelial cells. These findings contribute to a better knowledge of EV-mediated communication amongst cancer cells and between cancer cells and peritoneal mesothelial cells, which in turn is crucial to understanding the pathogenesis of OvC and CRC peritoneal metastasis.

## 2. Results

### 2.1. Characterization of EVs Derived from CRC Colo-320 and OvC SKOV-3 Cells

**Figure 1:**
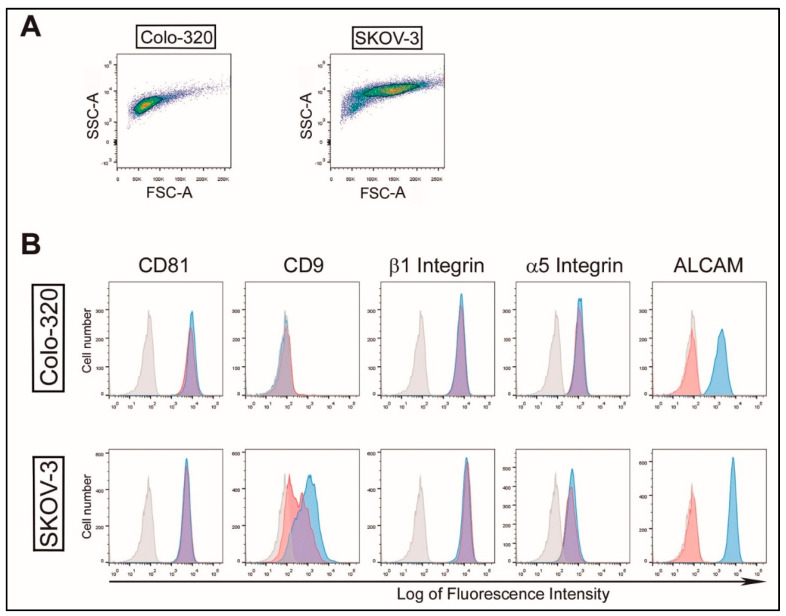
ALCAM surface expression on parental (wild-type) and CRISPR/Cas9 ALCAM-KO Colo-320 and SKOV-3 cells. (**A**) Representative gating strategies for flow cytometry analysis of Colo-320 and SKOV-3 cells, using the gates shown on forward scatter area (FCS-A) versus side scatter area (SSC-A) plots. (**B**) Flow cytometry detection of CD81, CD9, β1 integrin, α5 integrin and ALCAM on the surface of Colo-320 (colorectal adenocarcinoma) and SKOV-3 (ovarian adenocarcinoma) cells. Expression of molecules on parental (wild-type) cells are shown in blue profiles, whereas their respective ALCAM-KO cells (ALCAM-KO/Colo-320 and ALCAM-KO/SKOV-3) are shown in red profiles. The flow cytometry negative controls are shown in light-grey profiles.

To study the involvement of ALCAM/CD166 in the interactions and ensuing uptake of EVs produced by OvC (SKOV-3) and CRC (Colo-320) cells, we first suppressed the expression of this molecule in both cancer cell lines by using the CRISPR/Cas9 gene knock out technique. As shown in Figure 1, ALCAM/CD166 is abundantly expressed on the surface of Colo-320 and SKOV-3 parental (“wild-type”) cells but its expression is completely abrogated on CRISPR/Cas9 ALCAM-KO cells. In contrast, the expression of other cell surface molecules, such as the tetraspanin CD81 and integrin subunits α5 or β1, remained unaffected in ALCAM-KO cells compared to the wild-type counterparts. CD9 is not endogenously expressed on wild-type Colo-320 cells [36,37,38] and, as expected, the lack of expression of this tetraspanin was not affected upon CRISPR/Cas9 ALCAM KO in these cells. In contrast, the expression of CD9 on ALCAM-KO/SKOV-3 cells was greatly diminished compared to wild-type SKOV-3 cells, which indicates an interdependence between ALCAM and CD9 expression on these cells.

**Figure 2:**
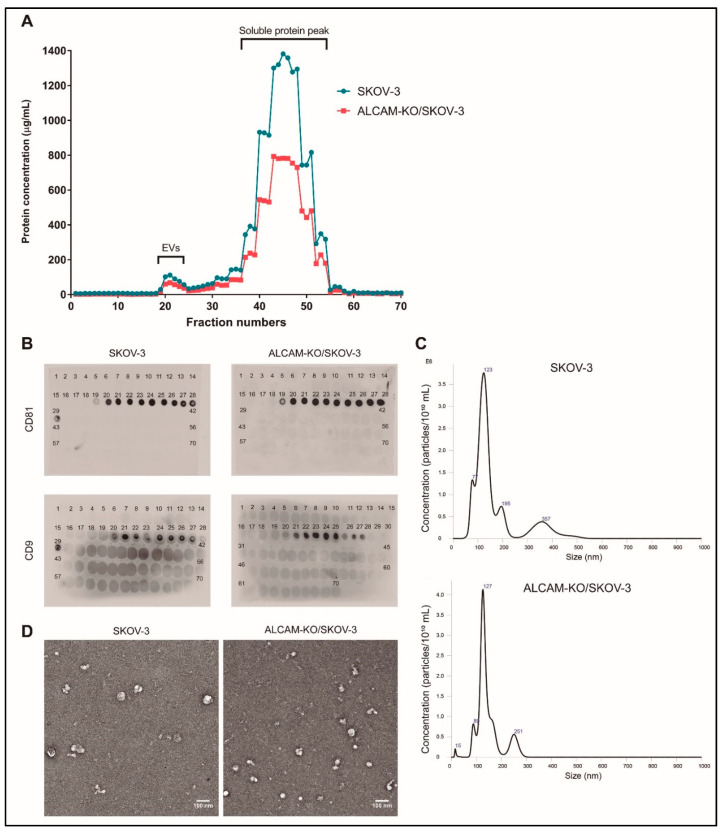
Characterization of SKOV-3 and ALCAM-KO/SKOV-3 EVs. (**A**) A representative profile of protein content (determined by Micro-BCA method) of the 70 fractions collected from the size exclusion chromatography (SEC) column. (**B**) Expression of CD9 or CD81 determined by Dot-Blot in the SEC eluted fractions from the conditioned media of SKOV-3 and ALCAM-KO/SKOV-3 cells. (**C**) Nano Tracking Analysis (NTA) of the size and concentration of SKOV-3 and ALCAM-KO/SKOV-3 EVs. (**D**) Transmission electron microscopy of negatively-stained EVs isolated from the conditioned media of SKOV-3 and ALCAM-KO/SKOV-3 cells. Scale bar = 100 nm.

Next, we isolated EVs produced by wild-type and ALCAM-KO/SKOV-3 cells by using a protocol involving the sequential concentration of EVs from FBS-free conditioned culture media with Tangential Flow Filtration (TFF) and spin filtration and subsequent fractionation using Size Exclusion Chromatography (SEC), as described previously in detail for Colo-320-derived EVs [36]. On average, a total of 70 fractions (0.5 mL each) were collected from each sample and their protein content was quantified using the Micro BCA Protein Kit. Figure 2A shows a representative graph of the protein content of all collected fractions from cultures of both wild-type and ALCAM-KO/SKOV-3 cells. Similar elution profiles were obtained with samples from cultures of Colo-320 cells, as reported by our group previously [36]. The following two protein peaks were consistently distinguishable in all elution profiles: a small first peak, which corresponds to the elution of EVs, typically comprising fractions of 21–27, and a much larger second peak (fractions 31–55) corresponding to the elution of free soluble protein. The EV content of the fractions in the first peak was confirmed with a dot blot analysis using antibodies against the tetraspanins CD81 and CD9, two classical EV markers (Figure 2B) [39,40].

The CD81/CD9-positive fractions of the first peak (fractions 21–27) were pooled and the size distribution and concentration of EVs contained in the resulting samples were determined with Nanoparticle Tracking Analysis (NTA) (Figure 2C). Typically, the EV concentration in pooled samples was in the range 3.5–4.0 × 10^10^ particles/mL, and the diameter of the majority of particles fell within the range of 70–150 nm, with the highest peak at around 125 nm, as expected for small EVs. The morphology and size of these particles, as visualized by transmission electron microscopy after uranile negative staining, also corresponded to sEVs (Figure 2D). Using the same protocol for the isolation of EVs, we recently reported the detection of CD81, TSG101 and β1 integrin using Western blot in EVs isolated from the conditioned medium of Colo-320 cells [36], further confirming that the EVs contained in fractions 21–27 are indeed bona fide sEVs [40].

### 2.2. ALCAM/CD166 Mediates Interactions of Tumor-Derived EVs with Recipient Cancer Cells

**Figure 3:**
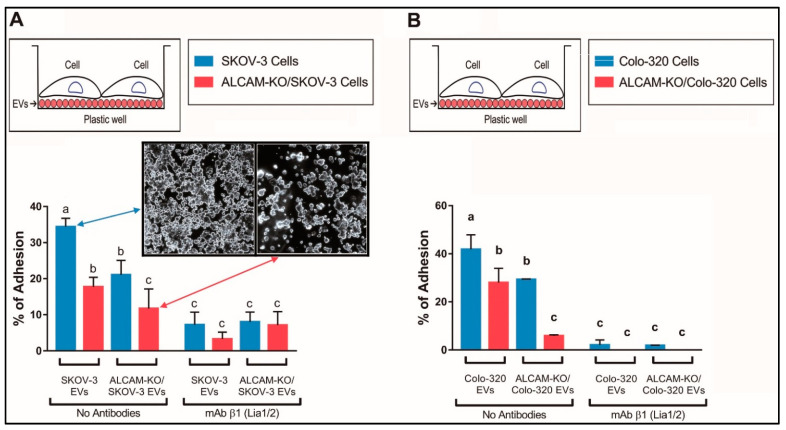
The absence of ALCAM from either cells or EVs reduces cell adhesion to immobilized EVs. SKOV-3 cell lines, either wild-type (blue bars) or ALCAM-KO (red bars) and Colo-320 cell lines, either wild-type (blue bars) or ALCAM-KO (red bars) were adhered to their respective cell-derived EVs (produced by either wild-type or ALCAM-KO cells), that had been previously immobilized on a 96-well plate. Adhesion of SKOV-3 and ALCAM-KO/SKOV-3 (**A**), and Colo-320 and ALCAM-KO/Colo-320 cells (**B**) to immobilized EVs was reduced when ALCAM was absent from either the cells or the EVs. For both cell lines, the reduction in cell adhesion was even higher when ALCAM was absent from both the cells and the EVs. The insets in (**A**) show photographs of wild-type SKOV-3 cells adhered to EVs produced by wild-type SKOV-3 cells (left inset) and of ALCAM-KO SKOV-3 cells adhered to EVs produced by ALCAM-KO SKOV-3 cells (right inset). Adhesion assays were also performed in the presence of the anti-β1 integrin mAb Lia1/2, that blocks the interaction of this subset of integrins with their ligands. Adhesion assays were performed as described in the Materials and Methods. The percentage of cells that remained adhered after washing out non-adherent cells is indicated for each condition as mean ± SEM of three experiments, each performed in duplicate wells. The statistical analysis performed was a two-way ANOVA. The letters above each bar (a, b, c) denote significant differences amongst the different conditions (*p* ≤ 0.05), while different bars with the same letter indicate that the differences amongst them are not statistically significant.

To determine the involvement of ALCAM/CD166 in the interactions between cancer-derived EVs and tumor cells, EVs isolated from parental SKOV-3 cells (“SKOV-3 EVs”, expressing abundant ALCAM) or from ALCAM-KO/SKOV-3 cells (“ALCAM-KO/SKOV-3 EVs”, lacking ALCAM expression) were first immobilized on plastic wells. The ability of these two types of EVs to support the adhesion of either parental or ALCAM-KO/SKOV-3 cells was then quantified by determining the percentage of fluorescently labeled cells that remained bound to the EV-coated wells after washing out the non-bound cells. Figure 3A shows that the capacity of SKOV-3 cells lacking ALCAM expression (ALCAM-KO/SKOV-3 cells) to adhere to “SKOV-3 EVs” was greatly diminished (18% adherent cells) compared to that of parental ALCAM-expressing SKOV-3 cells (35% adherent cells), clearly indicating the involvement of cellular ALCAM in EV–cells interactions. Furthermore, the capacity of “ALCAM-KO/SKOV-3 EVs” to support the adhesion of SKOV-3 cells was also greatly reduced (21% adherent cells) compared to that of ALCAM-expressing “SKOV-3 EVs” (35% adherent cells), indicating that ALCAM on the EV surface is also involved in the interactions between cancer cells and EVs. A further reduction in cell adherence (12% adherent cells) was observed when ALCAM was absent from both interacting partners, cells (“ALCAM-KO/SKOV-3 cells”) and EVs (“ALCAM-KO/SKOV-3 EVs”), confirming the relevance of ALCAM expression on cells and EVs.

Since several β1 integrins were previously reported by our group [36] and others [41,42] to play a role in the interactions between cancer-derived EVs and recipient target cells, we assessed the involvement of members of this subfamily of adhesion molecules by employing an anti-β1 integrin blocking monoclonal antibody (mAb Lia 1/2). The data show that, in addition to ALCAM, β1 integrins are also involved in the interactions between SKOV-3 cells (either expressing ALCAM or not) and SKOV-3 EVs (either expressing ALCAM or not), since in all cases the presence of mAb Lia1/2 reduced the percentages of cell adhesion to immobilized EVs (Figure 3A).

To exclude the possibility that the observed involvement of ALCAM in the interactions between SKOV-3 cells and the SKOV-3-derived EVs was a particularity of this ovarian carcinoma cell line and their EVs, we also carried out similar adhesion experiments with the colorectal carcinoma Colo-320 cells (either expressing ALCAM or not) and EVs derived from them (either expressing ALCAM or not). The results of these experiments (Figure 3B) established that ALCAM is also involved in the adhesion of Colo-320 cells to immobilized EVs, providing support for a more general role of this adhesion molecule in the interactions between cancer cells and EVs derived from them.

**Figure 4:**
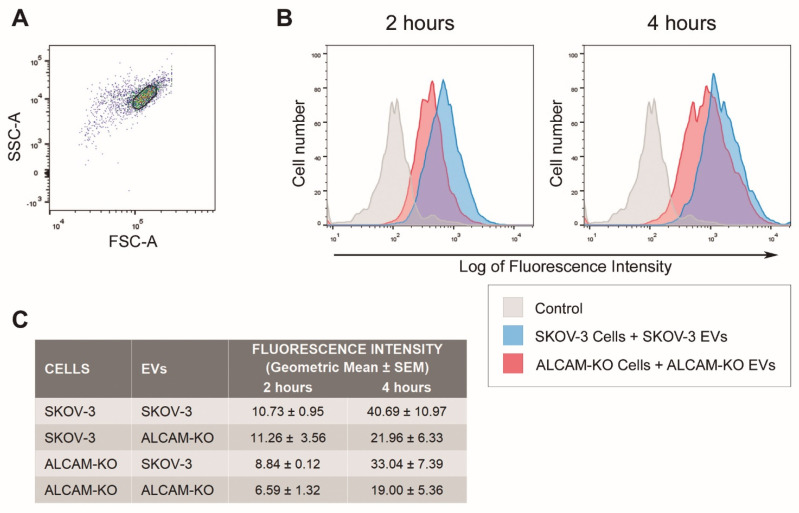
Involvement of ALCAM in EV uptake by cancer cells shown by flow cytometry. Adherent SKOV-3 and ALCAM-KO/SKOV-3 cells were incubated with AlexaFluor™ 633-C5-maleimide-stained EVs for 2 h or 4 h, and EV uptake was quantified with flow cytometry as described in the Materials and Methods. (**A**) Representative gating strategy for flow cytometry analysis of the SKOV-3 cells, using the gate shown on the forward scatter area (FCS-A) versus side scatter area (SSC-A) plot. (**B**) SKOV-3 cells internalized more efficiently SKOV-3-derived EVs (blue profiles) than ALCAM-KO/SKOV-3 cells internalized ALCAM-KO/SKOV-3-derived EVs (red profiles), at both incubation times. (**C**) Quantitative measurement of EV uptake for all cells-EVs combinations tested, at 2 h and 4 h. The presented values were obtained from the flow cytometry analysis and represent the means of fluorescence intensity values (each normalized to their respective negative controls) ± SEM of three experiments.

After their engagement in initial receptor/ligand-mediated interactions with recipient cells, EVs are generally subsequently taken up and internalized in the cell cytoplasm, where they deliver their biomolecular cargo that is responsible for the phenotypic and functional effects induced in the target cells [3,41,43]. Therefore, we investigated whether ALCAM, in addition to supporting interactions of EVs with recipient cells, was also relevant in the subsequent exosomal uptake. For this purpose, we quantitatively assessed, using flow cytometry, the uptake of fluorescently labeled SKOV-3-derived EVs (either expressing or lacking ALCAM) by both wild-type SKOV-3 and ALCAM-KO/SKOV-3 cells. Figure 4B shows that the uptake of SKOV-3-derived EVs was markedly reduced when ALCAM was absent both from the surface of recipient cells (ALCAM-KO/SKOV-3 cells) and from EVs (ALCAM-KO/SKOV-3 EVs), compared to the uptake of wild-type (ALCAM-expressing) EVs by wild-type (ALCAM-expressing) cells. The reduced uptake was evident both after 2 h and 4 h of co-incubating EVs with cells (Figure 4A). Interestingly, as quantitatively assessed using the geometric mean fluorescence values provided by the flow cytometry analysis (Figure 4C), EV uptake was also diminished when ALCAM was only absent from one side of the two interacting partners—either absent from ALCAM-KO/SKOV-3 cells or from ALCAM-KO/SKOV-3-derived EVs—though the inhibition levels did not reach those observed when ALCAM was absent from both sides.

**Figure 5:**
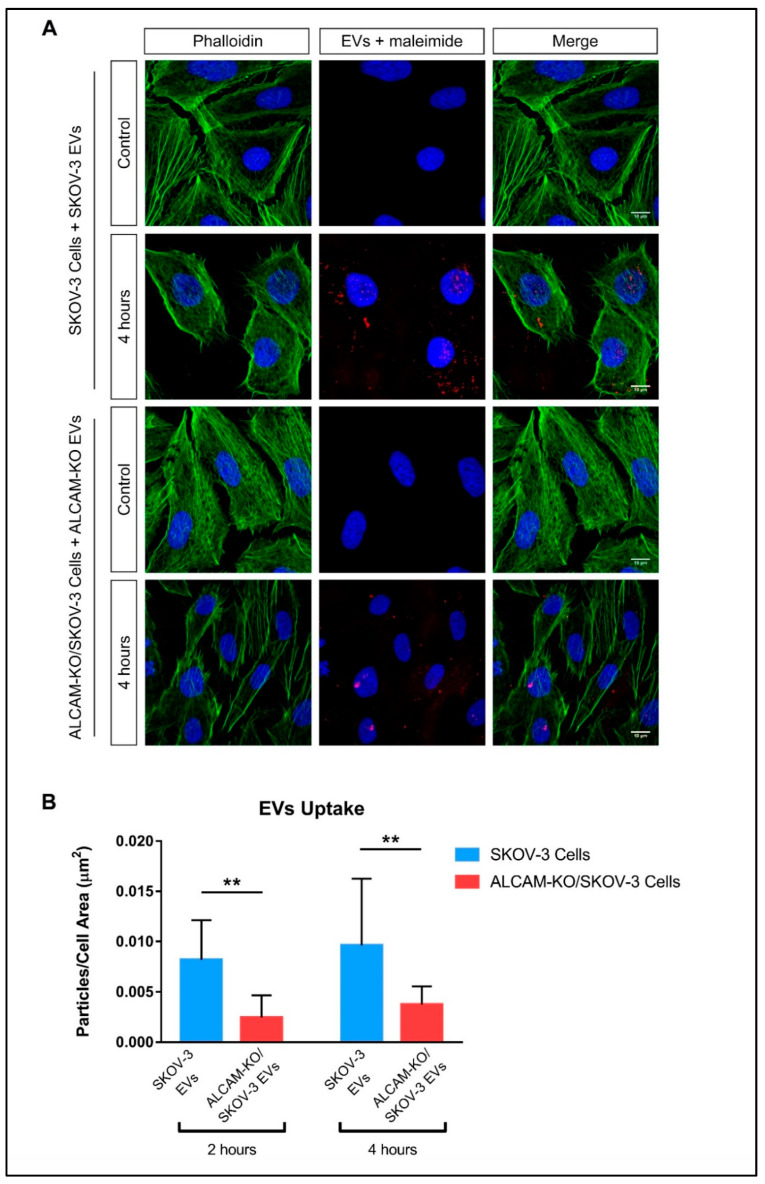
Involvement of ALCAM in EV uptake by cancer cells shown by confocal microscopy. (**A**) Confocal microscopy images showing the uptake of ALCAM+ and ALCAM− EVs by SKOV-3 and ALCAM-KO/SKOV-3 cells. Cells were incubated for 4 h with EVs that had been previously stained with AlexaFluor™ 633-C5-maleimide or with unstained EVs (control conditions). Scale bars in images = 10 µm (**B**) Quantitative determination of EV uptake for SKOV-3 cells and ALCAM-KO/SKOV-3 cells to their derived maleimide-stained-EVs after 2 and 4 h of incubation. The bar graphs show the quantification of the stained particles present in individual cells, expressed as particles/cell area (µm^2^). Statistical analysis performed was two-way ANOVA. ** *p* < 0.01.

We also confirmed, using confocal microscopy, that ALCAM-expressing wild-type SKOV-3 cells take up EVs more efficiently than ALCAM-KO/SKOV-3 cells (Figure 5). Of note, although the limit of the resolution of conventional confocal microscopy is above the size of the EVs employed here (around 120 nm), the fluorescent signals detected in these images likely correspond to aggregates of EVs that accumulate in endosomal compartments after being taken up by cells, evidenced by their colocalization with the tetraspanin CD63, as previously reported by our group [36].

**Figure 6:**
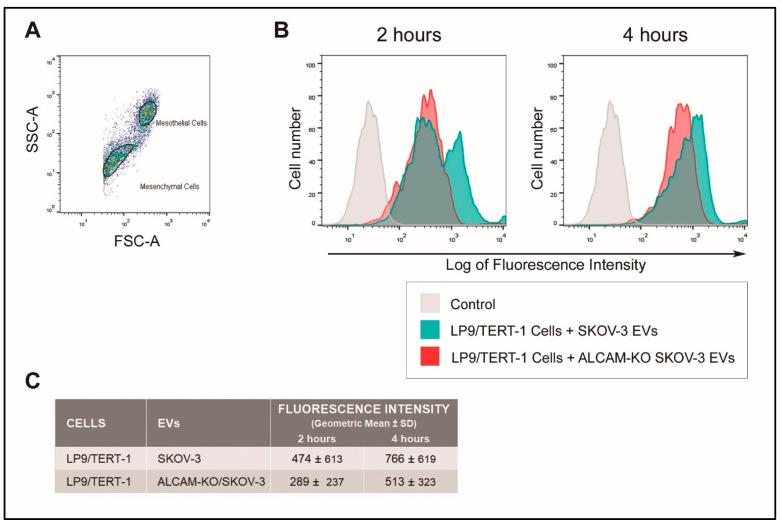
Involvement of ALCAM in EV uptake by peritoneal mesothelial cells. Adherent immortalized human peritoneal mesothelial LP9/TERT-1 cells were incubated with AlexaFluor™ 633-C5-maleimide-stained EVs produced by wild-type or ALCAM-KO SKOV-3 cells for 2 or 4 h and EV uptake was quantified by flow cytometry as described in Materials and Methods. (**A**) LP9/TERT-1 cells display heterogenous morphological features characteristic of mesothelial and mesenchymal cells [46]. The “mesothelial” cell population was gated (upper gate) on the forward scatter area (FCS-A) versus side scatter area (SSC-A) plot for subsequent analysis of EV uptake by flow cytometry. (**B**) Mesothelial LP9/TERT-1 cells internalized more efficiently EVs produced by wild-type SKOV-3 cells (green profiles) than EVs produced by ALCAM-KO/SKOV-3 cells (red profiles), at both incubation times. (**C**) Quantitative measurement of EV uptake by mesothelial LP9/TERT-1 cells, at 2 and 4 h time points. The presented values were obtained from the flow cytometry analysis and represent the geometric mean of fluorescence intensity values of a representative experiment.

In OvC and CRC pathogenesis, EVs released by cancer cells do not only mediate communication amongst cancer cells, but they also interact with—and are taken up by— peritoneal mesothelial cells (PMCs), in which they induce important phenotypic and functional changes that are associated with peritoneal metastasis. Therefore, we assessed the involvement of ALCAM/CD166 in the uptake of EVs produced by wild-type and ALCAM-KO SKOV-3 cells by human mesothelial LP9/TERT-1 cells, an immortalized cell line that phenotypically and functionally resembles normal human PMCs [44,45]. Figure 6 shows that EVs produced by ALCAM-expressing (wild-type) SKOV-3 cells are taken up by LP9/TERT-1 mesothelial cells more efficiently than EVs without ALCAM (produced by ALCAM-KO/SKOV-3 cells). These results indicate that ALCAM on the surface of cancer-derived EVs is also involved in EV uptake by mesothelial cells.

**Figure 7:**
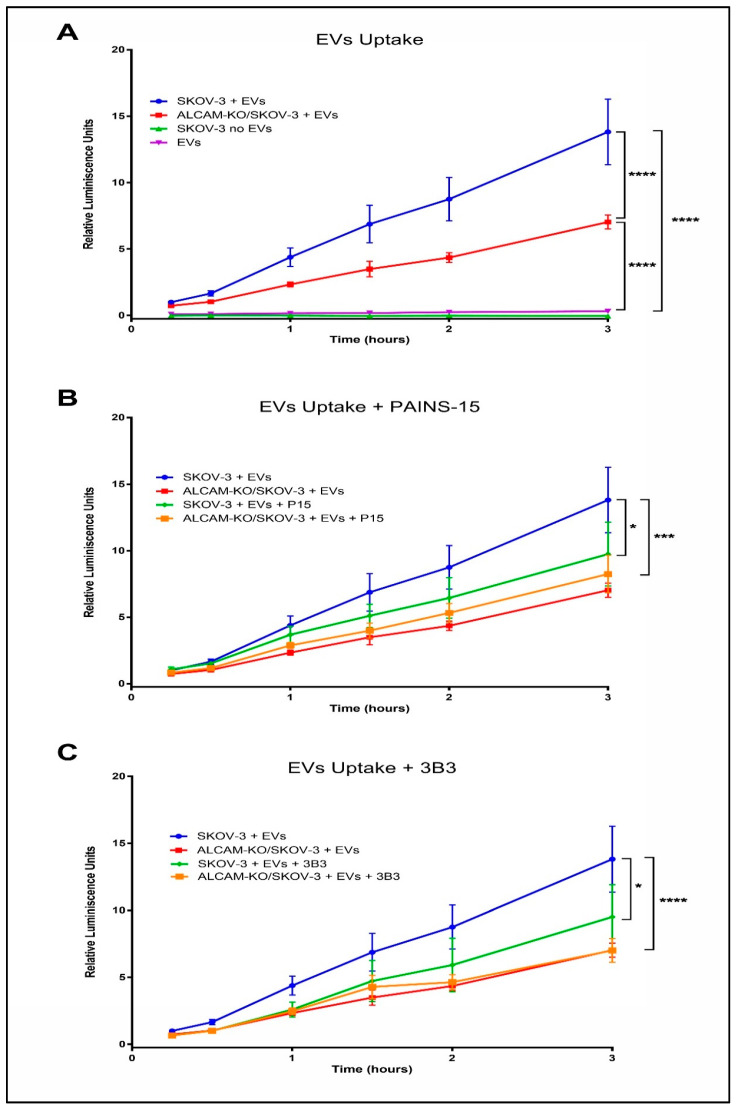
Anti-ALCAM mAbs impair EV uptake. SKOV-3 (blue lines) and ALCAM-KO/SKOV-3 (red lines) recipient cells were incubated with EVs derived from breast cancer SUM159 cells stably transfected with the DSP1-CD9 and DSP2 components of the EGFP/Renilla luciferase split protein [47]. EV uptake was assessed from luciferase luminescence detection, as previously described [47]. EV uptake was measured in the absence of an anti-ALCAM mAb (**A**), or in the presence of the anti-ALCAM mAb PAINS-15 (**B**), or the anti-ALCAM mAb 3B3 (**C**). Data are represented as Relative Luminiscence Units (RLUs) normalized to RLUs from the 0.25h time point of the SKOV-3 + EVs condition. Statistical analysis performed was two-way ANOVA. * *p* < 0.05, *** *p* < 0.001, **** *p* < 0.0001.

Our group recently reported a method that allows for the sensitive and quantitative measurement of the uptake of EVs that contain a fully reconstituted split dual EGFP-Renilla luciferase protein, leading to fluorescence and bioluminescence detection. These EVs are produced by human breast cancer SUM159 cells stably transfected with the two components of the split protein system (DSP1-coupled to the tetraspanin CD9 for optimal expression at the EV membrane, and DSP2) and permits the discrimination of EV uptake by recipient cells from EV binding to the cell surface thanks to the use of Enduren^TM^ (Promega Biotech, Alcobendas, Spain), a cytopermeable chemically protected Renilla luciferase substrate that only becomes the real substrate after the action of intracellular sterases [47]. We decided to assess whether the ALCAM involvement in EV uptake by cancer cells, as evidenced in the above-described flow cytometry and confocal microscopy experiments, could be confirmed using these dual EGFP-Renilla luciferase-containing EVs produced by SUM159 cells. ALCAM-KO/SKOV-3 cells (lacking ALCAM expression) showed a statistically significantly reduced capacity to take up EVs produced by SUM159 cells, compared to wild-type SKOV-3 cells (expressing ALCAM) (Figure 7A). Furthermore, the presence of different blocking anti-ALCAM mAbs (mAb PAINS-15, Figure 7B; mAb 3B3, Figure 7C) during the uptake experiment also led to a statistically significant reduction in the amount of EVs taken up by recipient wild-type SKOV-3 cells (which express ALCAM), but had no effect on the EV uptake by ALCAM-KO/SKOV-3 cells, thus ruling out an off-target effect of these antibodies.

## 3. Discussion

In OvC and CRC pathogenesis, tumor-derived EVs promote peritoneal metastasis through their specific interaction with—and uptake by—different immune and non-immune target cells in the peritoneal environment, in which they induce crucial phenotypic and functional changes that are involved in the metastatic process. In this regard, tumor-derived EVs facilitate the direct attachment of cancer cells to peritoneal mesothelial cells (PMCs) that line all peritoneal organs and structures, thus promoting the invasion of cancer cells through the mesothelial barrier. On the other hand, tumor-derived EVs induce the conversion of PMCs into cancer-associated fibroblasts (CAFs), which in turn, favor the survival, proliferation, and invasion of cancer cells, contributing to the metastatic process (reviewed in [12,13,14,15]). Importantly, tumor-derived EVs also mediate communication amongst cancer cells themselves, leading to the acquisition of a more aggressive and invasive phenotype, as well as conferring chemotherapy resistance [16,17,18,19].

While the promoting role of EVs in OvC and CRC peritoneal metastasis has been relatively well established, the identification of molecules involved in the multiple interactions between tumor-derived EVs and immune and non-immune peritoneal target cells remains rather incomplete. Relatedly, different adhesion molecules belonging to the integrin (including subunits αL/CD11a, αM/CD11b, αX/CD11c, β2/CD18, α4/CD49c, α5/CD49d, β1/CD29, αV/CD51, β3/CD61), immunoglobulin (ICAM-1/CD54) and selectin (CD62L, CD62P) families, as well as CD44 and the tetraspanins that associate with those adhesion molecules and organize them into Tetraspanin-Enriched Microdomains (including CD9, CD81, CD151), have been so far proposed to participate in EV-target cell interactions (reviewed in [2,6,48,49]). In most cases, the involvement of these molecules in EV interactions and in the ensuing cellular uptake was inferred from the inhibitory effects of blocking monoclonal antibodies. Of utmost importance, the specific repertoire of integrins (including members α6β4, α6β1 and αvβ5) expressed on the EVs produced by breast and pancreatic cancer cells has been shown to dictate their interactions with—and uptake by— specific target resident cells (lung fibroblasts, liver Kupffer cells), thus ultimately determining the lung or liver metastasis organotropism. Furthermore, it was proposed that integrins on EVs are not only involved in the interactions of these vesicles but also in triggering signaling pathways and inflammatory responses in recipient cells, thereby resulting in the “education” of the target organ, thus rendering it permissive for the growth of metastatic cells. However, the potential involvement of these or additional EV-integrins in dictating the interactions and uptake of EVs produced by OvC and CRC cells with specific immune and non-immune peritoneal target cells is currently not known. On a similar note, our group recently reported that the interaction between cellular integrin α5β1 and ADAM17 on EVs participates in the binding and uptake of CRC EVs by recipient PMCs and cancer cells, which is relevant in the process of peritoneal dissemination [36].

In the current research, we isolated the EVs produced by Colo-320 (colorectal adenocarcinoma) and SKOV-3 (ovarian adenocarcinoma) human cell lines and employed them as a model system to study the involvement of the adhesion molecule ALCAM/CD166 in EV interactions with—and in their subsequent cellular uptake by—cancer cells and by PMCs. As stated above, tumor-derived EVs represent an important vehicle of communication amongst cancer cells in mediating the acquisition of a more invasive and/or chemoresistant phenotype, which is a hallmark of theses cancers with peritoneal dissemination [16,17,18,19]. On the other hand, the interactions (and uptake) of tumor-derived EVs with PMCs are also of crucial relevance in the process of peritoneal metastasis, as they facilitate the invasion of cancer cells through the mesothelial barrier and the conversion of PMCs into CAFs, which further fuels the metastatic process [12,13,14,15].

To study the specific involvement of ALCAM/CD166, the expression of this molecule was knocked-out in SKOV-3 and Colo-320 cancer cells by using the CRISPR/Cas9 gene disruption technology. EVs were isolated from both the parental wild-type cells (producing ALCAM+ EVs) and from the ALCAM-KO cell lines (producing ALCAM-EVs). By comparing the differential capacity of immobilized ALCAM+ and ALCAM− EVs to support the adhesion of wild-type (ALCAM+) and ALCAM-KO (ALCAM−) SKOV-3 and Colo-320 cells, we established that ALCAM/CD166 is involved in EV interactions with recipient cancer cells. By using a blocking monoclonal antibody specific to the β1 integrin subunit (mAb Lia1/2), we confirmed that, in parallel to ALCAM-mediated interactions, members of the β1 integrin subfamily are also involved in EV interactions with cancer cells. These results are in agreement with previous reports published by our group [36] and others [41,48,50], showing the implication of β1 integrins in the docking and uptake of cancer-derived EVs.

Interestingly, we observed that the interactions between EVs and cells (evidenced by the quantification of cell adherence to immobilized EVs) were further reduced when ALCAM was absent from both interacting partners (i.e., cells and EVs) compared to cell adherence when ALCAM was only absent from either cells or from EVs, which would indicate that, in addition to ALCAM–ALCAM homophilic interactions, this adhesion molecule may engage in heterophilic interactions with alternative ligands expressed on cells and on EVs. Likewise, the subsequent EV uptake was also reduced when ALCAM was absent from either interacting partners, cells or EVs, and again, the reduction in EV uptake was more pronounced when ALCAM was absent from both sides. Again, these results indicate that besides its engagement in homophilic interactions, ALCAM involvement in EV uptake may also implicate heterophilic interactions with other ligands. In this regard, to date, the best characterized ligand that has been reported to engage in heterophilic interactions with ALCAM is the co-stimulatory molecule CD6. However, the expression of CD6 is relatively restricted to lymphoid cells and we confirmed, using flow cytometry, that CD6 is not expressed on the surface of SKOV-3 or Colo-320 cells (data not shown), ruling out the involvement of CD6-ALCAM heterophilic interactions in EV docking and uptake in our experimental setting.

In addition to CD6, several other extracellular and intracellular molecules (including Galectin-8, SOSTDC1, S100B, CD9, and Ezrin) have been reported as ligands/interactors for ALCAM, although these heterophilic interactions have not been characterized as extensively as that of ALCAM with CD6 [reviewed recently in [28]]. With the possible exception of the tetraspanin CD9, to our knowledge, the implication of any of these molecules in the interactions and uptake of EVs by recipient target cells remains completely unexplored. Some years ago, our group reported the direct association of ALCAM with CD9 in protein complexes on the cell surface that also include the metalloproteinase ADAM17/TACE [32,37,49]. The expression of CD9 in those protein complexes enhanced both the homophilic (ALCAM-ALCAM) and heterophilic (ALCAM-CD6) ALCAM interactions through a dual mechanism involving the augmented clustering of ALCAM molecules and upregulation of ALCAM surface expression due to the inhibition of ADAM17/TACE sheddase activity. Interestingly, we observed that the expression of CD9 on ALCAM-KO/SKOV-3 cells was greatly diminished compared to that on wild-type SKOV-3 cells, which further supports the relevance of a direct CD9-ALCAM association on the cell surface. This finding could be explained by different and non-mutually exclusive possibilities, including (i) that ALCAM could act as a CD9 chaperone, which is required for its traffic towards the cell surface; (ii) that ALCAM is required for maintaining a stable expression of CD9 on the cell surface by inhibiting its endocytosis and subsequent entry in a cellular degradative route. In this regard, we have observed an increased number of lysosomes in ALCAM-KO/SKOV-3 cells compared to wild-type SKOV-3 cells (data not shown), suggestive of an enhanced cellular degradative activity. Furthermore, knocking-out CD9 in SK-MEL-147 melanoma cells also resulted in an increased number of lysosomes [51], supporting the notion that the association of ALCAM and CD9 may constitute a functional unit in cancer cells.

In a recent article, we stated that the neo-expression of CD9 on the EV surface negatively regulates the interactions and uptake of Colo-320 cells-derived EVs with recipient target cells, by inhibiting the adhesive capacity of ADAM17, which acts as an EV ligand for cellular integrin α5β1 [36]. The mechanism by which CD9 negatively regulates the adhesive capacity of ADAM17 could be related to changes induced by CD9 in the organization and size of discrete clusters of this metalloproteinase, which would negatively impinge on the overall avidity of adhesion [37]. Therefore, different molecules on the EV surface (including ALCAM and ADAM17) are involved in the interactions between tumor-derived EVs and recipient cells and the adhesive capacity of these molecules may be subjected to additional regulation by EV CD9.

**Figure 8:**
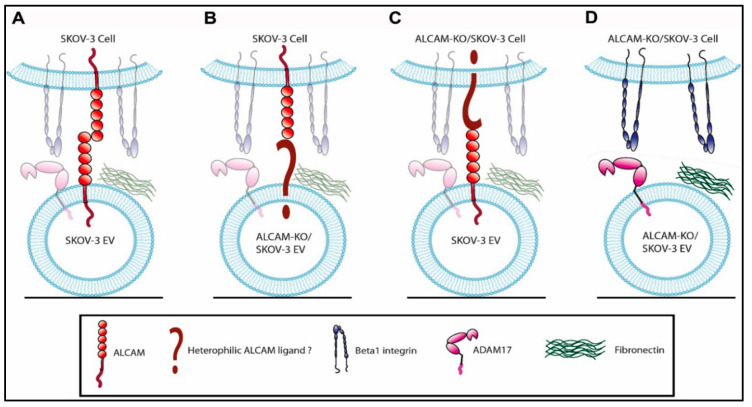
Schematic representation of the involvement of ALCAM and β1 integrin in the interactions between cancer-derived EVs and cancer cells. When ALCAM molecules are expressed both on EV and cell surfaces, they can mediate the binding and subsequent uptake of cancer derived EVs through homophilic ALCAM-ALCAM interactions (**A**). ALCAM expressed on the cell surface can engage in heterophilic interactions with an unidentified ligand expressed on the surface of EVs lacking ALCAM (**B**). ALCAM expressed on the EV surface can engage in heterophilic interactions with an unidentified ligand expressed on the surface of cells lacking ALCAM (**C**). β integrins expressed on the surface of cells lacking ALCAM expression can mediate the binding and uptake of cancer-derived EVs lacking ALCAM through their interactions with specific EV ligands, such as ADAM17 or fibronectin (**D**).

We have provided, in Figure 8, a schematic representation of the different possibilities of ALCAM and β1 integrin involvement in the interactions between cancer-derived EVs and cancer cells, based on the data presented here. When ALCAM molecules are expressed both on EV and cell surfaces, they can mediate the binding and subsequent uptake of cancer-derived EVs through homophilic ALCAM-ALCAM interactions (Figure 8A). ALCAM expressed on the cell surface can engage in heterophilic interactions with an unidentified ligand expressed on the surface of EVs lacking ALCAM (Figure 8B). ALCAM expressed on the EV surface can engage in heterophilic interactions with an unidentified ligand expressed on the surface of cells lacking ALCAM (Figure 8C). β integrins expressed on the surface of cells lacking ALCAM expression can mediate the binding and uptake of cancer-derived EVs lacking ALCAM through their interactions with specific EV ligands, such as the ADAM17 or fibronectin (Figure 8D).

In conclusion, we established that ALCAM/CD166 is involved in cancer-derived EV interactions with recipient cancer and peritoneal mesothelial cells and in their subsequent cellular uptake by these cells. We also confirmed the results of previous reports indicating that β1 integrins also participate in these processes. We believe that the identification of ALCAM/CD166 and β1 integrins as adhesion molecules that mediate the docking and uptake of CRC and OvC-derived EVs may contribute to the development of new therapeutic approaches aimed at blocking the peritoneal metastasis cascade promoted by EVs in CRC and OvC patients.

## 4. Materials and Methods

### 4.1. Cells and Antibodies

Human ovarian adenocarcinoma SKOV-3 cells (purchased from ATCC, cell line HTB-77) were cultured in medium DMEM (GIBCO, Madrid, Spain) supplemented with 10% heat-inactivated FBS (GIBCO, Madrid, Spain), 2 mM glutamine (Sigma-Aldrich Merck, Madrid, Spain), 50 μg/mL streptomycin (Sigma-Aldrich Merck, Madrid, Spain), and 50 U/mL penicillin (Sigma-Aldrich Merck, Madrid, Spain). Human colorectal adenocarcinoma Colo-320 cells (kindly provided by Dr. Nancy Hogg, Francis Crick Institute, London, UK) were cultured in medium RPMI-1640 (Sigma-Aldrich Merck, Madrid, Spain) supplemented with 10% heat-inactivated FBS, 2 mM glutamine, 50 μg/mL streptomycin and 50 U/mL penicillin. Breast cancer cell line SUM159 (kindly provided by Dr. Miguel Vicente-Manzanares, Center for Cancer Research, Salamanca, Spain) was cultured in z DMEM/F-12 (GIBCO, Madrid, Spain) culture medium supplemented with 5% FBS, 50 μg/mL streptomycin and 50 U/mL penicillin, non-essential amino acids (80 mg/mL) (HyClone GE Healthcare), insulin (5 μg/mL) and hydrocortisone (1 μg/mL). The immortalized human peritoneal mesothelial cell line LP9/TERT-1 (kindly provided by Dr. James G. Rheinwald, Boston, MA, USA), was cultured in Earle’s M199 medium (Thermo Fischer, Madrid, Spain) supplemented with 20% heat-inactivated FBS, 2 mM glutamine, 50 μg/mL streptomycin, 50 U/mL penicillin and 2% Biogro-2 (Biological Industries, Beit HaEmek, Israel).

To generate ALCAM-KO/SKOV-3 and ALCAM-KO/Colo-320 cell lines, cells were transfected with the CRISPR/Cas9 knockout plasmid pX461 encoding GFP and Cas9 nickase and the following sequences to generate the specific single-guide RNAs: 5′-CACCGAGTGCTTTGCTTACAATTTC-3′ and 5′-CACCGCGAAACAGAGCAGCT-AAAAA-3′. Transfected cells were sorted by flow cytometry based on their GFP transient fluorescence and then expanded and checked for the suppression of ALCAM expression.

The monoclonal antibodies used in this research were purified by protein A- or protein G-affinity chromatography, according to their isotype as described previously [52]. These antibodies included mAb 5A6 (anti-CD81 tetraspanin, kindly provided by Dr. Shoshana Levy, Stanford University, CA, USA) [53], mAb Lia1/2 (anti-β1 integrin; kindly provided by Dr. Francisco Sánchez-Madrid, Hospital de la Princesa, Madrid, Spain) [54,55], commercial mAbs TS2/16 (anti-β1 integrin, Santa Cruz Biotechnology sc-53711) P1D6 (anti-α5 integrin, Abcam ab78614), mAbs (generated and characterized in our laboratory) PAINS-10 (anti-CD9 tetraspanin), PAINS-15 (anti-ALCAM) and 3B3 (anti-ALCAM) [32,52].

### 4.2. Flow Cytometry

For flow cytometry, 2 × 10^5^ SKOV-3 or Colo-320 cells were incubated with the respective primary antibodies, anti-CD81 (5A6), anti-CD9 (PAINS10), anti-β1 integrin (TS2/16), anti-α5 integrin (P1D6) and anti-ALCAM (PAINS-15) for 30 min at 4 °C, washed three times in FBS-free RPMI-1640 and incubated with the secondary polyclonal antibody Alexa Fluor™ 647-conjugated Goat anti-mouse IgG (Thermo Fisher, Madrid, Spain) for 30 min at 4 °C. Cells were washed three times and then fixed in a 2% formaldehyde solution in PBS. Fluorescence was measured using a FACScan™ flow cytometer (Beckton-Dickinson, Madrid, Spain). The cytometry data were processed using the FlowJo (v10 version) software (BD Biosciences, Ashland, OR, USA).

### 4.3. EVs Purification

EVs were isolated as described previously for the Colo-320 cell line [36]. Briefly, SKOV-3 and Colo-320 were seeded at 1 × 10^6^ cells/mL, cultured and deprived of FBS for 72h before the culture medium was collected and centrifuged at 300× *g* for 5 min to eliminate cells and large debris, and at 10,000× *g* for 45 min to eliminate microvesicles and larger EVs. Then, the supernatant was concentrated to a volume of 45 mL by tangential flow filtration using a Vivaflow^®^ 50R, 10,000 MWCO membrane (Sartorius, Goettingen, Germany), and further concentrated using a 100 kDa Amicon Ultra-15 Centrifugal filter (Merck, Darmstadt, Germany) to a final volume of 1 mL, which was loaded onto a 25 mL size-exclusion chromatography (SEC) column (Sepharose 4 Fast-Flow resin) and eluted with filtered PBS. Typically, a total of 70 fractions (0.5 mL each) were collected from each sample.

### 4.4. Dot Blot

The presence of CD81 and CD9 tetraspanins in all fractions collected from SEC was assessed by Dot-blot. From each fraction, 2 μL were spotted onto a nitrocellulose membrane (Pall Life Science, Portsmouth, UK). Membranes were blocked with 3% BSA and incubated with primary mAb 5A6 (anti-CD81) and PAINS-10 (anti-CD9) (1 µg/mL), followed by three washes with 0.1% Tween20-TBS (TBS-T). The membranes were incubated with polyclonal secondary antibody HRP-conjugated Goat anti-mouse IgG (Sigma-Aldrich Merck, Madrid, Spain), followed by three washes with 0.1% TBS-T. Finally, they were incubated using the ECL Plus Western blotting Detection System (GE Healthcare, Madrid, Spain) solution and ECL-chemiluminescence detected in an ImageQuant LAS 4000 biomolecular imager (GE Healthcare).

### 4.5. Nano Tracking Analysis (NTA)

The size and the concentration of the SEC-purified EVs were assessed in a Nanosight NS500 platform (Malvern Instruments Ltd., Malvern, UK), after diluting the samples 1/100 in filtered PBS 1X. A total of three 1 min videos were acquired for each sample.

### 4.6. Transmission Electron Microscopy

For Transmission Electron Microscopy, EVs were negatively stained with 2% uranyl acetate in double-distilled water for 45 s. EVs were visualized with the Jeol JEM-1010 transmission electron microscope and the images were acquired with the 4KI 4K CMOS camera, F416 of TVIPS. The images acquired using the microscope were processed using the ImageJ (1.51j8 version) software [56].

### 4.7. Cell Adhesion Assays

Cell adhesion assays to EVs produced by Colo-320 and SKOV-3 cell lines were performed as previously described [36]. The wells of a 96-multi well flat bottom plate were coated with EVs (5 μg exosomal protein per well) overnight at 4 °C. Cells were stimulated with phorbol ester PMA (200 ng/mL) for 1 h and then loaded with the fluorescent probe BCECF-AM for 20 min at 37 °C in PBS. Cells were washed, incubated in 10% FBS-RPMI for 10 min at 37 °C and then resuspended in the adhesion buffer (20 mM Hepes, 150 mM NaCl, 2 mg/mL glucose, 1 mM MgCl2 and 0.5 mM CaCl_2_). For adhesion assays with Colo-320 cells, 1.5 × 10^5^ cells suspended in 100 µL of adhesion buffer were added to each well in the presence or absence of the blocking mAbs (all at 20 μg/mL, final concentration) anti-β1 integrin (Lia1/2), anti-α5 integrin (PID6) or anti-human fibronectin (HFN7.1). For adhesion assays with SKOV-3 cells, the 5 × 10^4^ cells suspended in 100 µL of adhesion buffer were added to each well in the presence or absence of the respective blocking mAbs. Cells were left to adhere to immobilized EVs for 40–60 min at 37 °C before being gently washed (3–5 washes) with 37 °C pre-warmed adhesion buffer. The percentage of adherent cells in each well was calculated by determining their fluorescence relative to the 100% input fluorescence (before removing the non-adherent cells) in a microplate reader (TecanGENios). All conditions (presence or absence of mAbs) in every experiment were performed in duplicate wells.

### 4.8. Measurement of Cellular EV Uptake

For the flow cytometry measurement of EV uptake, EVs (100 μg/100 µL) were incubated with AlexaFluor™ 633-C5-maleimide (0.05 mM) (Thermo Fisher Scientific) overnight at 4 °C. To remove the excess of maleimide, EVs were centrifuged in EV spin columns (with a 3000 MW cut-off) (Invitrogen, Madrid, Spain), according to the manufacturer’s instructions. Then, 7 μL of eluted labeled EVs were incubated with adhered 1.0 × 10^5^ SKOV-3, ALCAM-KO/SKOV-3 or LP9/TERT-1 cells in a final volume of 150 µL for 2 or 4 h. Cells were washed and treated with the trypsin-EDTA (0.25–0.02%) solution for 5 min in order to detach the SKOV-3, ALCAM-KO/SKOV-3 or LP9/TERT-1 cells from the plates and to remove EVs bound to the cell surface but not yet taken up by the cells. The fluorescence corresponding to the maleimide-labeled internalized EVs was measured in a FACScan™ flow cytometer (Beckton-Dickinson). The cytometry data were analyzed using the FlowJo (v10 version) software.

For the confocal microscopy assessment of EVs uptake, adhered 1.0 × 10^5^ SKOV-3 or ALCAM-KO/SKOV-3 cells, grown on glass coverslips, were incubated with 7 μL of eluted labeled EVs for 2 or 4 h. Cells were then washed and fixed with 1% formaldehyde in PBS for 8 min, and blocked in 3% BSA-TBS for 30 min before incubation with Phalloidin AlexaFluor™ 488 (Thermo Fisher Scientific) for 30 min. Cells were washed with TBS-T to remove the excess Phalloidin, and the coverslips were mounted on microscope slides with Fluoromount™/DAPI (Sigma-Aldrich Merck, Madrid, Spain). Images were acquired with the LSM710 vertical confocal microscope (Zeiss, Oberkochen, Germany). The quantification of the fluorescent dots in microscope images corresponding to the stained-EVs was performed using the ImageJ (1.51j8 version) software. Flourescent particles were only considered if they the following criteria: several adjacent pixels with fluorescence intensity ≥70 (in an intensity scale of 0–255), minimum size of 0.08 µm^2^, and 0.4 as minimum circularity. The number of particles present in each cell was normalized to the respective total cell area.

Measurement of luciferase-containing EVs uptake was performed as described [47]. Human breast cancer SUM159 cells stably expressing a DSP1-CD9/DSP2 construct were cultured for 5 days in the EV-depleted media until reaching confluence. Conditioned media from SUM159 DSP1-CD9/DSP2 cells was harvested and centrifuged at 400× *g*, 5 min to remove the remaining cells, at 4000× *g*, for 30 min to eliminate cellular debris and then at 100,000× *g* for 2 h at 4 °C to pellet the EVs. The EVs pellet was resuspended in 800 μL of filtered PBS. 3 × 10^3^ SKOV-3 or ALCAM-KO/SKOV-3 cells were cultured in the EV-depleted culture media in a clear bottom 96-multiwell plate for 24 h before being loaded with luciferase substrate Enduren (Promega) by incubating them for 2 h with 100 μL/well of the 60 μM Enduren solution in EV-depleted culture media. Supernatant was removed and cells were washed once with fresh EV-depleted media, in order to remove Enduren remnants. Around 4–8 μg of DSP1-CD9/DSP2-carrying EVs was added in a total volume of 50 μL diluted in EV-depleted media. Then, 3B3 or PAINS-15 anti-ALCAM blocking antibodies were added to a total volume of 100 μL to a final concentration of 10 μg/mL. Bioluminiscence was measured at different time points with a Tecan GENios Microplate reader, with 5000 ms of exposure time, leading to a gain of 150. The luciferase luminiscence signal, measured in Relative Luminiscence Units (RLU) was normalized to the RLU of the SKOV-3 cells + EVs treatment group, that was used as a control.

### 4.9. Statistical Analyses

Statistical analyses of data were performed using the GraphPad PRISM (7.0 version) software (GraphPad Software, San Diego, CA, USA). The analyses included a two-way ANOVA. The different levels of *p*-value considered for statistical significance are indicated by asterisks in the individual figure legends (* *p* < 0.05, ** *p* < 0.01, *** *p* < 0.001, **** *p* < 0.0001), or with different letters (a, b, c) in the legend of Figure 3, indicating a *p*-value ≤ 0.05.

## Data Availability

Any data or materials that support the findings of this study can be made available by the corresponding author upon request.
